# Transcriptional response of mar, sox and rob regulon against concentration gradient carbapenem stress within *Escherichia coli* isolated from hospital acquired infection

**DOI:** 10.1186/s13104-020-04999-2

**Published:** 2020-03-19

**Authors:** Shiela Chetri, Bhaskar Jyoti Das, Deepshikha Bhowmik, Debadatta Dhar Chanda, Atanu Chakravarty, Amitabha Bhattacharjee

**Affiliations:** 1 Assam University, Silchar, India; 2 Silchar Medical College and Hospital, Silchar, India

**Keywords:** marA, soxS, rob, Real-time PCR, *Escherichia coli*, Carbapenems

## Abstract

**Objective:**

The present study was carried out to investigate the transcriptional response of marA (Multiple antibiotic resistance A gene), soxS (Superoxide S gene) and rob (Right-origin-binding gene) under carbapenem stress.

**Results:**

12 isolates were found over-expressing AcrAB-TolC efflux pump system and showed reduced expression of OmpF (Outer membrane porin) gene were selected for further study. Among them, over expression of *marA* and *rob* was observed in 7 isolates. Increasing pattern of expression of *marA* and rob against meropenem was observed. The clones of *marA* and rob showed reduced susceptibility towards carbapenems.

## Introduction

Bacteria are known to be adapted against antimicrobial agents by means of acquired resistance determinants and several intrinsic resistance mechanisms like decreased cell permeability and increased efflux of the toxic agents [1–5]. Global regulators control these activities making them to survive against adverse conditions [6]. It is observed that marA, soxS and rob are activator of AcrAB-TolC tripartite efflux pump systems. Over expression of these regulators has resulted in multidrug resistant phenotype [7–12]. Carbapenems are considered to be the last therapeutic option for all gram-negative infections [1, 13] hence, it is imperative to know how these global transcriptional regulators respond when they are exposed to carbapenems. Also, till now no such report has predicted or established their role in carbapenem resistance, instead they are found to be responsible for tetracycline, chloramphenicol, ampicillin, nalidixic acid, and rifampin resistance [8]. These multiple antibiotic resistance regulators pose a potential threat to future therapeutic outcome. Global transcriptional regulators are also known to be involved in stress response in bacteria. As carbapenem resistance is in an increasing trend in hospital acquired infections, the present study was carried out to observe transcriptional response of *marA*, *soxS* and *rob* against concentration dependent carbapenem stress.

## Main text

### Methodology

#### Bacterial sample

A total of 198 consecutive, non-duplicates, *Escherichia coli* isolates were selected for the study. These isolates were collected from clinical samples obtained from Silchar Medical College and Hospital, Silchar, India between June 2014 and May 2015. *E. coli* isolates were selected based on their non-susceptibility to at least one of the carbapenem and *E. coli* ATCC 25922 was used as the quality control strain.

#### Transcriptional expression of AcrAB-TolC and ompF

To analyse the expressional level of efflux pump genes *acrA* and *acrB* in multidrug resistant clinical isolates of *Escherichia coli* quantitative Real Time PCR was performed. For Real Time PCR, total cellular RNA was isolated using the Qiagen Rneasy Mini Kit (Qiagen, Germany) according to the manufacturer’s instructions. cDNA was prepared by using Qiagen Reverse Transcription Kit (QIAGEN, Germany) and was quantified by Picodrop (Pico200, Cambridge, UK). Further, Real Time PCR amplification was performed using power Sybrgreen PCR master mix reagents kit (Applied Biosystems, Austin, USA) and the expression levels of *acrA* and *acrB* were assessed using StepOnePlus quantitative Real Time-PCR (Applied Biosystems, USA) using oligonucleotide primers [acrA (F): 5′CTCTCAGGCAGCTTAGCCCTAA3′, acrA (R): 5′TGCAGAGGTTCAGTTTTGACTGTT3′)] [15], [acrB (F): 5′AGCTTCCTGATGGTTGTCGG3′, acrB (R): 5′ACGGCTGATGGCATCTTTCA3′, [Omp F (F):5′AAGTAGTAGGTTGCGCCCAC3′, OmpF (R): 5′AGTTCGATTTCGGTCTGCGT3′]. The experiment was performed by using a house keeping gene *RpslE* as an internal control and the relative C_t_ value of the target genes were compared with that of the control *E. coli* ATCC 25922 to determine the fold change in the expressional level of mRNA of the test isolates. Each sample was processed in triplicates.

#### Transcriptional expression of marA, soxS and rob

Isolates with over expressed AcrAB-TolC were selected for this experiment and total RNA was isolated using Qiagen RNase Mini Kit (Qiagen, Germany), reverse transcribed into cDNA by using QuantiTect^®^ reverse transcription kit (Qiagen, Germany). Quantification of cDNA was done by Pico drop (Pico 200, Cambridge, UK) and quantitative real time PCR was performed using Power SYBR Green Master Mix (Applied Biosystems, Warrington, UK) in StepOnePlus Real Time PCR (Applied Biosystems, USA) using primers for amplification of marA, soxS and rob genes as listed in Additional file 1: Table S2. The house keeping gene *rpseL* of *E. coli* was used as an internal standard. And the relative expression of the targeted genes was determined by ΔΔCt method. Expression analysis was carried out to measure the relative expression of the mRNA compared with that of *E. coli* ATCC 25922. Each sample was processed in triplicates.

#### Determination of transcriptional expression of the local regulator acrR gene

Isolates over-expressing AcrAB and AcrADefflux pump systems were selected and the transcriptional expression of the local regulatory gene AcrR were demonstrated by quantitative Real Time PCR using primers (forward primer: 5′ACAAGAAGCGCAAGAAACGC3′ and reverse primer: 5′CCAGCGAGGTGGATGATACC3′). *E. coli* ATCC 25922 was used as a reference strain. Transcriptional response of AcrR against concentration gradient carbapenem stress was also analysed by Real time PCR assay.

#### Transcriptional response of marA, soxS and rob under concentration gradient carbapenem stress

To test the effect of carbapenems on global transcriptional regulators, *E. coli* isolates were exposed to sub-inhibitory concentrations of meropenem, ertapenem and imipenem ranging from 0.25 µg/ml to 2 µg/ml. RNA was extracted using an Qiagen RNase Mini Kit (Qiagen, Germany) followed by cDNA synthesis using QuantiTect^®^ reverse transcription kit (Qiagen, Germany) as per manufacturer’s instructions. Quantitative Real Time PCR was performed with specific primers (Additional file 1: Table S2) as per described earlier. Each sample was processed in triplicates and their relative expression was compared with that of *E. coli* ATCC 25922.

#### Sequencing of marA, soxS and rob

To detect any mutation in regions known to be involved in the regulation marA, soxS, and rob was amplified by using primers (Additional file 1: Table S3). The PCR products were sequenced using Sanger’s method. Sequences were compared with those from the GenBank nucleotide database using the Basic Local Alignment Search Tool (http://www.ncbi.nlm.nih.gov).

#### Cloning of marA, soxS and rob

The global regulatory genes were amplified as mentioned earlier (Additional file 1: Table S3) for *marA, soxS* and *rob*. PCR amplification was performed using 50 μl of total reaction volume. The PCR products were then confirmed by 1.0% (w/v) agarose gels and purified using the Qiaquick^®^ Gel Extraction Kit (Hilden, Germany) and cloned into pGEM -T vector (Promega, Madison, USA). The resulting recombinant plasmids were transformed into *E. coli* DH5α by heat shock method for functional characterization. Antimicrobial susceptibility testing of the clones were done by Kirby Bauer disc diffusion method against carbapenem antibiotics i.e. meropenem (10 µg), ertapenem (10 µg) and imipenem (10 µg). Minimum inhibitory concentration of the clones against carbapenems was determined via agar dilution method. The results were interpreted as per CLSI 2017 guidelines [16].

### Statistical analysis

The differences in relative expression of efflux pump gene regulatory genes *marA, soxS* and *rob* was compared with that of the wild type strain (both under normal condition and under concentration gradient carbapenem stress) between samples were determined with the help of one-way ANOVA followed by Tukey–Kramer (Tukey’s W) multiple comparison test. Differences were considered statistically significant at both 5% and 1% level when p < 0.05. SPSS version 17.0 was used for statistical analysis.

## Result

**Fig. 1:**
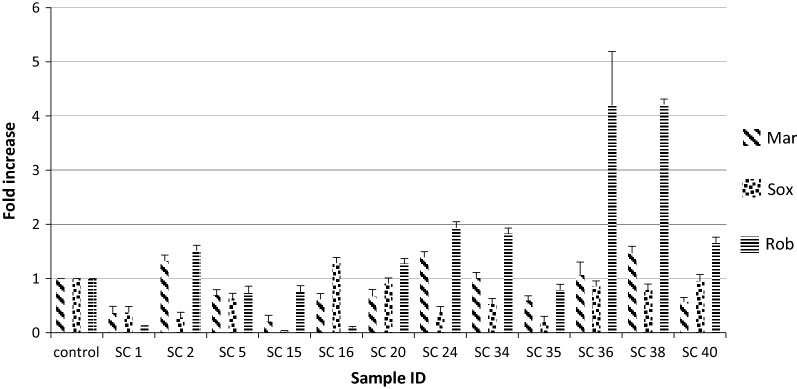
Expression of Mar, Sox and Rob of AcrAB overexpressing isolates under normal condition (without stress) relative *Escherichia coli* ATCC 25922

**Fig. 2:**
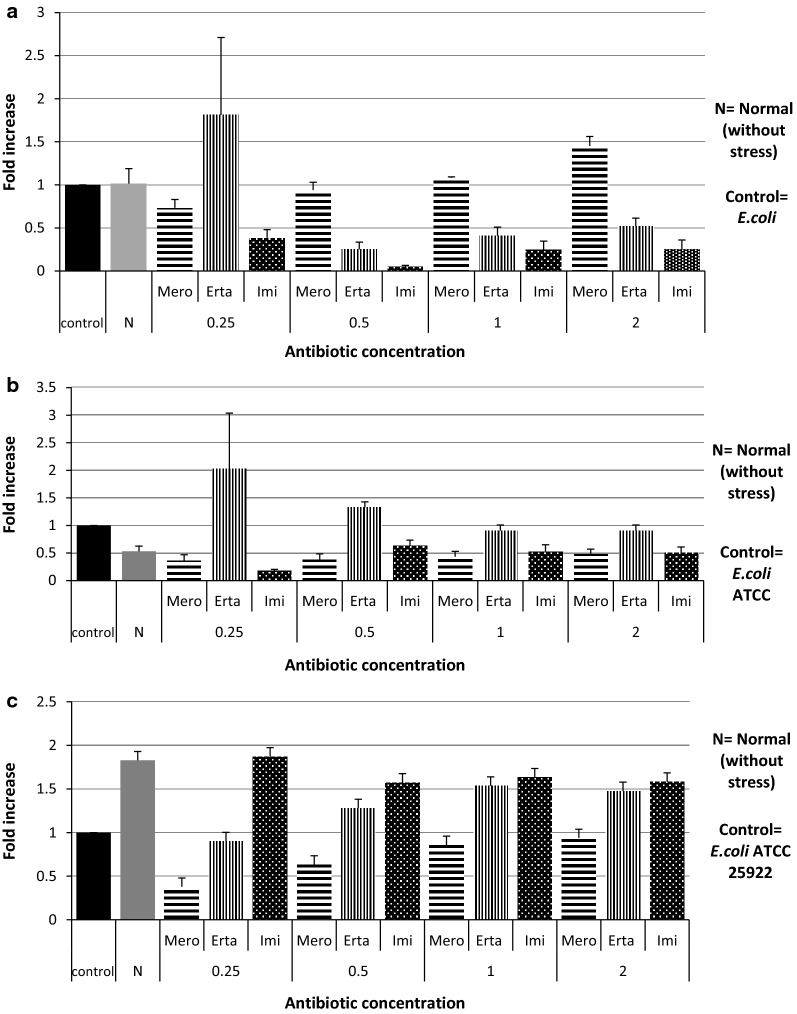
Expression of MarA (**a**), SoxS (**b**) and Rob (**c**) gene under carbapenem stress relative to *Escherichia coli* ATCC 25922

**Fig. 3:**
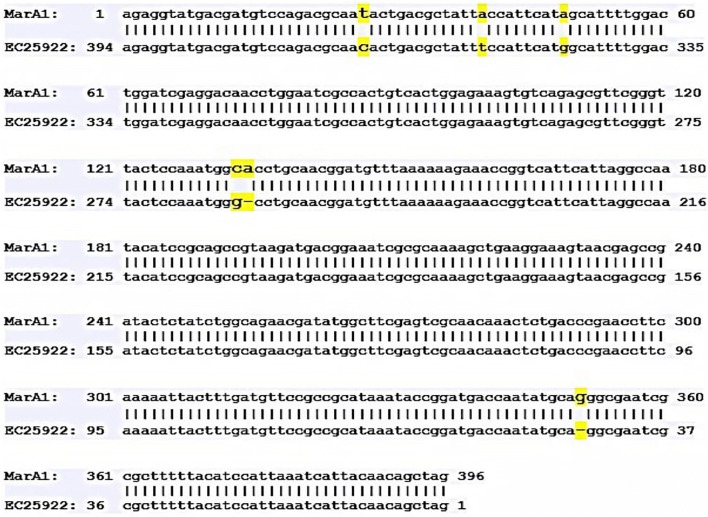
Sequence alignment of mutational pattern of marA sequence with the sequence of *Escherichia coli* ATCC 25922 strain

Out of 198 carbapenem non-susceptible *E. coli* isolates, 44.94% (89/198) were found to be resistant towards at least one of the carbapenems tested and were devoid of any carbapenemase genes. Of them 12 exhibited *AcrAB*-*TolC* over expression and down regulation of OmpF, were further selected (Additional file 1: Table S1). While analysing the transcriptional expression, more than half of them (n = 7) showed down regulation of *marA*, and similar trend was too observed for *SoxS* regulon where six isolates showed downregulation. However, in case of *rob*, more than half of isolates (n = 7) showed over expression (Fig. 1). To determine whether carbapenem exposure confers any change in the transcriptional expression of the *marA, soxS* and *rob* genes quantitative Real Time PCR was done and a substantial escalation in the expression level of *marA* against meropenem was seen (Fig. 2a). However, against ertapenem and imipenem stress the expression of *marA* was not consistent. In case of *soxS*, a similar trend of expression pattern was observed irrespective of concentration gradient meropenem stress where as, transcriptional expression was inversely proportional with increasing concentration of ertapenem stress (Fig. 2b). Towards imipenem the expression level of *soxS* displayed an inconsistent pattern. Correspondingly, the expression level of rob showed a steady increase towards increasing concentration of meropenem (Fig. 2c), while towards ertapenem and imipenem concentration gradient exposure there was an unsystematic pattern in the expression. Further, when the DNA sequences of *marA, soxS* and *rob* were compared with the reference strain of *E. coli* ATCC 25922 it displayed nucleotide alterations at many locations. We observed four-point mutations in 27th (t-c), 40th (a-t) 49th (a-g) and 133rd (c-g) position and 2 deletion mutations in the 134th and 351st position in *marA* (Fig. 3). However, no observable mutations in the sequence of *rob* and *soxS* was noticed. MIC range and antimicrobial susceptibility pattern of the clones showed that the zone of inhibition of transformants (mar and rob) was decreased as compared with that of parent strain (DH5α) as well as with the plasmid without gene of interest (Additional file 1: Table S4). Further, MIC range of the clones were determined of which two-fold or more increase in the inhibitory concentration against ertapenem and imipenem was noted for clone of *rob* and *mar* while comparing with the parent strain (DH5α) (Additional file 1: Table S4).

## Discussion

Carbapenems are the last line antibiotics available to the world and this investigation is an approach to get a better understanding on the role of global transcriptional regulators towards carbapenem non-susceptibility which may be helpful to find out a way to identify new targets for antimicrobials. This study excludes all the carbapenemase producers to solely concentrate on a particular resistance mechanism against carbapenems. Efflux pump plays a major role in conferring resistance towards several antimicrobial agents as reports on efflux pump, AcrAB-TolC system showing resistance against many compounds like dyes, detergents including various classes of antibiotics [14]. However, in response to external stress bacteria have the ability to adjust their own mechanisms by regulating gene network by transcriptional machinery [1]. These global regulators marA [15, 17, 18], soxS [19] and rob [20, 21] exhibit multiple antibiotic resistance phenotypes by activating AcrAB –TolC efflux pump system in *Escherichia coli*. In previous studies marA mediated tigecycline and imipenem resistant phenotype was observed [22, 23]. In the present study increase in marA expression in *E. coli* is found to be correlated with the over-expression of AcrAB efflux pump under concentration gradient meropenem stress. Other than marA, soxS also plays an important role in developing resistance in bacteria towards oxidative stress environment [24–26]. soxS and rob increases the expression of AcrAB efflux pump when induced with agents like paraquat and sodium decanoate (a bile salt) [27]. However, in the present study, the expression of soxS is increased in AcrAB overexpressed strains of *E. coli* when exposed under meropenem stress condition which is not been reported elsewhere. In an earlier study salicylate was reported to be capable of activating marRAB via rob [28]. It has also been reported that the overexpression of rob exhibits resistance against antibiotics, organic solvents and superoxide-generating agents through plasmid [16, 29]. In this study strong correlations between overexpression of rob and carbapenem resistance was observed when exposed to concentration gradient meropenem stress which is not being reported earlier. The overall patterns of the three regulators in this study showed that rob was significantly affecting carbapenem susceptibility. This result of the investigation underscores the ability of carbapenem antibiotics to induce the transcriptional expression of global transcriptional regulators which in turn would contribute in carbapenem resistance.

## Conclusion

Our study demonstrates the role of the global regulators *marA*, *soxS* and *rob* in triggering the overexpression of AcrAB efflux pump system conferring resistance towards carbapenems. The result of this investigation highlights the fact that the global regulators directly or indirectly involved in increased expression of the efflux pump system leading to the emergence of carbapenem resistant MDR *Escherichia coli* isolates in clinical settings.

## Limitation

Therefore, these global regulators marA, soxS and Rob play an important role in developing resistance towards the last resort carbapenem antibiotics which calls for further investigation.

## Supplementary information

**Additional file 1: Table S1.** MIC and transcriptional study details of the test isolates. **Table S2.** Primers used for PCR and Real time PCR. **Table S3.** Primers used for cloning. **Table S4.** Carbapenem susceptibility and MIC results of clones.

## Data Availability

All the data generated in this research work are presented in this research article. In case of any additional information requirement corresponding author will be providing the necessary information as per ethical guidelines.

## Abbreviations

Mar: Multiple antibiotic resistance
Sox: Superoxide
Rob: Right-origin-binding
cDNA: Complementary DNA
OmpF: Outer membrane porin F

## References

[1] Duvaland V, Lister IM (2013). MarA, SoxS and Rob of *Escherichia coli*—global regulators of multidrug resistance, virulence and stress response. Int J Biotech Well Indus..

[2] Yang Q, Wang H, Sun H, Chen H, Xu Y, Chen M (2010). Phenotypic and genotypic characterization of *Enterobacteriaceae* with decreased susceptibility to carbapenems: results from large hospital-based surveillance studies in China. Antimicrob Agents Chemother.

[3] Oteo J, Delgado-Iribarren A, Vega D, Bautista V, Rodríguez MC, Velasco M, Saavedra JM, Pérez-Vázquez M, García-Cobos S, Martínez-Martínez L, Campos J (2008). Emergence of imipenem resistance in clinical *Escherichia coli* during therapy. Int J Antimicrob Agents.

[4] Poirel L, Héritier C, Spicq C, Nordmann P (2004). In vivo acquisition of high-level resistance to imipenem in *Escherichia coli*. J Clin Microbiol.

[5] Stapleton PD, Shannon KP, French GL (1999). Carbapenem resistance in *Escherichia coli* associated with plasmid-determined CMY-4 β-lactamase production and loss of an outer membrane protein. Antimicrob Agents Chemother.

[6] Ishihama A (2010). Prokaryotic genome regulation: multifactor promoters, multitarget regulators and hierarchic networks. FEMS Microbiol Rev.

[7] Li XZ, Nikaido H (2004). Efflux-mediated drug resistance in bacteria. Drugs..

[8] Okusu H, Ma D, Nikaido H (1996). AcrAB efflux pump plays a major role in the antibiotic resistance phenotype of *Escherichia coli* multiple-antibiotic-resistance (Mar) mutants. J Bacteriol..

[9] Greenberg JT, Chou JH, Monach PA, Demple B (1991). Activation of oxidative stress genes by mutations at the soxQ/cfxB/marA locus of *Escherichia coli*. J Bacteriol.

[10] Nikaido H (1996). Multidrug efflux pumps of gram-negative bacteria. J Bacteriol..

[11] Alekshun MN, Levy SB (1997). Regulation of chromosomally mediated multiple antibiotic resistance: the mar regulon. Antimicrob Agents Chemother.

[12] Perez A, Poza M, Aranda J, Latasa C, Medrano FJ, Tomas M, Romero A, Lasa I, Bou G (2012). Effect of the transcriptional activators SoxS, RobA and RamA on expression of the multidrug efflux pump AcrAB-TolC in *Enterobacter cloacae*. Antimicrob Agents Chemother.

[13] Hsueh PR, Hoban DJ, Carmeli Y, Chen SY, Desikan S, Alejandria M, Ko WC, Binh TQ (2011). Consensus review of the epidemiology and appropriate antimicrobial therapy of complicated urinary tract infections in Asia-Pacific region. J Infect.

[14] Du D, Wang Z, James NR, Voss JE, Klimont E, Ohene-Agyei T, Venter H, Chiu W, Luisi BF (2014). Structure of the AcrAB–TolC multidrug efflux pump. Nature.

[15] Cohen SP, Hächler H, Levy SB (1993). Genetic and functional analysis of the multiple antibiotic resistance (mar) locus in *Escherichia coli*. J Bacteriol.

[16] CLSI. Performance standards for antimicrobial susceptibility testing. 27th ed. CLSI supplement M100. Wayne, PA: Clinical and laboratory standards institute; 2017.

[17] Rhee S, Martin RG, Rosner JL, Davies DR (1998). A novel DNA-binding motif in MarA: the first structure for an AraC family transcriptional activator. Proc Nat Acad Sci..

[18] Martin RG, Rosner JL (2001). The AraC transcriptional activators. Curr Opin Microbiol.

[19] Miller PF, Gambino LF, Sulavik MC, Gracheck SJ (1994). Genetic relationship between soxRS and mar loci in promoting multiple antibiotic resistance in *Escherichia coli*. Antimicrob Agents Chemother.

[20] Jair KW, Yu X, Skarstad K, Thöny B, Fujita N, Ishihama A, Wolf RE (1996). Transcriptional activation of promoters of the superoxide and multiple antibiotic resistance regulons by Rob, a binding protein of the *Escherichia coli* origin of chromosomal replication. J Bacteriol..

[21] Tanaka T, Horii T, Shibayama K, Sato K, Ohsuka S, Arakawa Y, Yamaki KI, Takagi K, Ohta M (1997). RobA-induced multiple antibiotic resistance largely depends on the activation of the AcrAB efflux. Microbiol Immunol.

[22] Veleba M, Higgins PG, Gonzalez G, Seifert H, Schneiders T (2012). Characterisation of RarA, a novel AraC-family multidrug resistance regulator in *Klebsiella pneumoniae*. Antimicrob Agents Chemother.

[23] Bornet C, Chollet R, Malléa M, Chevalier J, Davin-Regli A, Pagès JM, Bollet C (2003). Imipenem and expression of multidrug efflux pump in *Enterobacter aerogenes*. Biochem Biophys Res Commun..

[24] Wu J, Weiss BE (1991). Two divergently transcribed genes, soxR and soxS, control a superoxide response regulon of *Escherichia coli*. J Bacteriol..

[25] Amabile-Cuevas CF, Demple B (1991). Molecular characterization of the soxRS genes of *Escherichia coli*: two genes control a superoxide stress regulon. Nucleic Acids Res.

[26] Wu J, Weiss BE (1992). Two-stage induction of the soxRS (superoxide response) regulon of *Escherichia coli*. J Bacteriol..

[27] Chubiz LM, Glekas GD, Rao CV (2012). Transcriptional crosstalk within the mar/sox/rob regulon in *Escherichia coli* is limited to the rob and marRAB operons. J Bacteriol.

[28] Ariza RR, Li Z, Ringstad N, Demple B (1995). Activation of multiple antibiotic resistance and binding of stress-inducible promoters by *Escherichia coli* Rob protein. J Bacteriol.

[29] Nakajima H, Kobayashi K, Kobayashi M, Asako H, Aono R (1995). Overexpression of the robA gene increases organic solvent tolerance and multiple antibiotic and heavy metal ion resistance in *Escherichia coli*. Appl Environ Microbiol.

