# Supplementary material for: Transcriptional response of mar, sox and rob regulon against concentration gradient carbapenem stress within Escherichia coli isolated from hospital acquired infection
Source: BMC Res Notes. 2020 Mar 19;13:168. doi: 10.1186/s13104-020-04999-2 (PMC7083032; doi:10.1186/s13104-020-04999-2)
Supplement: Supplementary file 1 — Additional file 1: Table S1. MIC and transcriptional study details of the test isolates. Table S2. Primers used for PCR and Real time PCR. Table S3. Primers used for cloning. Table S4. Carbapenem susceptibility and MIC results of clones. [file 13104_2020_4999_MOESM1_ESM.docx]

**Table S1: MIC and transcriptional study details of the test isolates**

| Strain ID | MIC Range µg/ml | | | RQ value | | |
| --- | --- | --- | --- | --- | --- | --- |
|  | Meropenem | Ertapenem | Imipenem | AcrA | AcrB | OmpF |
| SC 1 | 2 | 4 | 2 | 1.907 (↑) | 7.853(↑) | 0.537(↓) |
| SC 2 | 128 | 128 | 128 | 2.143 (↑) | 3.778(↑) | 0.126 (↓) |
| SC 3 | ˃512 | 512 | 128 | 69.315 (↑) | 1.354(↑) | 0.024(↓) |
| SC 4 | 64 | 256 | 256 | 8.794 (↑) | 39.446(↑) | 0.52(↓) |
| SC 5 | 8 | 32 | 4 | 1.8 (↑) | 18.079(↑) | 0.083(↓) |
| SC 6 | 256 | ˃512 | 128 | 6.554 (↑) | 12.502(↑) | 0.464(↓) |
| SC 7 | 512 | ˃512 | ˃512 | 2.698 (↑) | 3.152(↑) | 0.225(↓) |
| SC 8 | 16 | 128 | 32 | 8.197 (↑) | 13.402(↑) | 0.411(↓) |
| SC 9 | 16 | 256 | 8 | 2.615 (↑) | 22.995(↑) | 0.041(↓) |
| SC 10 | 42 | 512 | 32 | 255.39 (↑) | 89.946(↑) | 0.617(↓) |
| SC 11 | 16 | 64 | 16 | 3.818 (↑) | 76.162(↑) | 0.281(↓) |
| SC 12 | 128 | 128 | 128 | 75.904 (↑) | 6.775(↑) | 0.035(↓) |

**Table S2. Primers used for PCR and Real time PCR.**

| *primer pairs* | *Target* | *Sequence (5′-3′)* | *Amplified Product Size (bp)* | *Reference* |
| --- | --- | --- | --- | --- |
| MarA- F  MarA- R | MarA | CTGGAATCGCCACTGTCACT  TTTGCGCGATTTCCGTCATC | 142 | This study |
| SoxS-F  SoxS-R | SoxS | GCGAGACATAACCCAGGTCC  TCAGACGCTTGGCGATTACA | 113 | This study |
| Rob- F  Rob-R | Rob | ACCAATCAGCGGCGTATCTT  TACCGCCGTTCTCCTGAATG | 114 | This study |

**Table S3. Primers used for cloning**

| *primer pairs* | *Target* | *Sequence (5′-3′)* | *Amplified Product Size (bp)* | *Reference* |
| --- | --- | --- | --- | --- |
| MarA- F  MarA- R | MarA | CAACACTTGAGCATTTGCTTAAG  GCAATCATGAAACCACTTTTATC | 490 | This study |
| SoxS-F  SoxS-R | SoxS | ATGGATGGGGTAATTACCCG  CTGTTCAGTTCGTTAATTCATCTGTT | 424 | This study |
| Rob- F  Rob-R | Rob | CTGGCTGGGTCGTTGAAACCGC  GGTAATTGGATAATAGCATT | 1000 | This study |

**Table S4: Carbapenem susceptibility and MIC results of clones**

| Sample ID | Antimicrobial susceptibility pattern | | | Minimum inhibitory concentration results | | |
| --- | --- | --- | --- | --- | --- | --- |
|  | Meropenem | Ertapenem | Imipenem | Meropenem | Ertapenem | Imipenem |
| DH5α | 5.7 | 5.6 | 3.8 | No growth | 0.125 | 0.125 |
| DH5α with intact plasmid | 4.7 | 4.8 | 3.6 | 0.125 | 0.125 | 0.125 |
| P^Mar^ | 4.3 | 3.7 | 3.3 | 0.125 | 0.5 | 0.25 |
| P^Rob^ | 3.6 | 3.8 | 3 | 0.25 | 0.5 | 0.5 |
